# Reply to Comment on Hardacker, C.T.; Baccellieri, A.; Mueller, E.R.; Brubaker, L.; Hutchins, G.; Zhang, J.L.Y.; Hebert-Beirne, J. Bladder Health Experiences, Perceptions and Knowledge of Sexual and Gender Minorities. Int. J. Environ. Res. Public Health 2019, 16, 3170

**DOI:** 10.3390/ijerph17072458

**Published:** 2020-04-04

**Authors:** Cecilia T. Hardacker, Anna Baccellieri, Elizabeth R. Mueller, Linda Brubaker, Georgia Hutchins, Jory Luc Yimei Zhang, Jeni Hebert-Beirne

**Affiliations:** 1 Howard Brown Health, Chicago, IL 60613, USA; ceciliah@howardbrown.org (C.T.H.); joryz@howardbrown.org (J.L.Y.Z.); 2 Rush University College of Nursing, Chicago, IL 60612, USA; 3 Community Health Sciences, School of Public Health, University of Illinois at Chicago, Chicago, IL 60612, USA; abacce1@uic.edu (A.B.); ghutch2@uic.edu (G.H.); 4 Departments of Obstetrics and Gynecology, Loyola University Stritch School of Medicine, Maywood, IL 60153, USA; emuelle@lumc.edu; 5 Departments of Urology, Loyola University Stritch School of Medicine, Maywood, IL 60153, USA; 6 Department of Obstetrics, Gynecology, and Reproductive Sciences, University of California San Diego, La Jolla, CA 92093, USA; librubaker@ucsd.edu

We appreciate your comments and concerns about our manuscript in IJERPH on Bladder Health Experiences, Perceptions and Knowledge of Sexual and Gender Minorities [1]. Our first response to you is to respectfully reiterate that this study was intended to collect, as the title of the publication states, experiences, perceptions and knowledge of bladder health from focus group (FG) participants, not to focus on symptomology. That being said, as evidenced in the data, we clearly collected plentiful information on participants’ bladder health symptoms as part of characterizing our sample. 

Without question, your description of methodologies to assess pathology and functionality are future considerations for sexual and gender minority (SGM) populations and your clearly articulated suggestions for clinical follow up are important. Our consortium consists of urologists and clinical researchers who agree that treatment modalities must be employed with this group in the future, yet your suggested in-depth course of treatment was not part of this community-based participatory research (CBPR) plan. Our clinical urology research team members were also concerned about the lost opportunity to intervene with the multiple bladder-LUTS issues raised in the FGs. In the design phase of this study, we struggled with ending each session by providing informational interventions such as local health resources and to potentially answer questions raised during the focus group. Ultimately we chose to provide broad national resource sheets about bladder health to participants.

Once again, we appreciate your interest in our CBPR exploration of bladder health with SGM populations and hope this addresses your comments. Thank you, Cecilia T. Hardacker, Anna Baccellieri, Elizabeth R. Mueller, Linda Brubaker, Georgia Hutchins, Jory Luc Yimei Zhang and Jeni Hebert-Beirne.
